# Reliability of Genetic Alterations in Predicting Ceftriaxone Resistance in Neisseria gonorrhoeae Globally

**DOI:** 10.1128/spectrum.02065-21

**Published:** 2022-03-29

**Authors:** Eric Yu Lin, Paul C. Adamson, Sung-min Ha, Jeffrey D. Klausner

**Affiliations:** a David Geffen School of Medicine at UCLA, Los Angeles, California, USA; b Division of Infectious Diseases, David Geffen School of Medicine at UCLA, Los Angeles, California, USA; c Department of Integrative Biology and Physiology, UCLA, Los Angeles, California, USA; d Department of Population and Public Health Sciences, Keck School of Medicine of USC, Los Angeles, California, USA; University at Albany, State University of New York

**Keywords:** *Neisseria gonorrhoeae*, algorithms, assays, ceftriaxone, antimicrobial resistance, genetic markers, genetic information database

## Abstract

Antimicrobial resistance in N. gonorrhoeae is increasing globally, and ceftriaxone is the recommended treatment for empirical therapy in most settings. Developing molecular assays to detect decreased ceftriaxone susceptibility is critical. Using PathogenWatch, a public database of N. gonorrhoeae genomes, antibiotic susceptibility data and DNA sequences of different genes associated with ceftriaxone resistance were extracted. That information was used to determine the sensitivity and specificity of different molecular markers and algorithms to predict decreased susceptibility to ceftriaxone. A total of 12,943 N. gonorrhoeae genomes were extracted from the PathogenWatch database, of which 9,540 genomes were used in the analysis. The sensitivity and specificity of specific molecular markers and algorithms were largely consistent with prior reports. Small variation (<10%) in either sensitivity or specificity occurred. Certain algorithms using different molecular markers at various prevalence of decreased ceftriaxone susceptibility identified a potentially clinically useful range of positive and negative predictive values. We validated previously described mutations and algorithms in a large public database containing a global collection of N. gonorrhoeae genomes. Certain mutations and algorithms resulted in sensitivity and specificity values consistent with those of prior studies. Further research is needed to integrate these markers and algorithms into the development of molecular assays to predict decreased ceftriaxone susceptibility.

**IMPORTANCE** Antimicrobial resistance in Neisseria gonorrhoeae (N. gonorrhoeae), the causative agent of gonorrhea, is rising globally. Ceftriaxone is the last remaining antibiotic for empirical treatment of gonorrhea. Developing molecular tests to predict ceftriaxone resistance can help to improve detection and surveillance of ceftriaxone resistance. Here, we utilized PathogenWatch, a public global online database of N. gonorrhoeae genomes, to evaluate different genetic markers in predicting decreased susceptibility to ceftriaxone. We compiled MICs for ceftriaxone from the PathogenWatch database and used a computational approach to extract all the genetic markers from the genomic data. We determined the sensitivity and specificity for predicting decreased ceftriaxone susceptibility among several combinations of genetic markers. We identified several combinations of genetic markers with high predictive values for decreased susceptibility to ceftriaxone. These combinations of genetic markers might be promising candidates for future molecular tests to predict ceftriaxone resistance.

## INTRODUCTION

Antimicrobial resistance in Neisseria gonorrhoeae is a major global health threat. As the second most common bacterial sexually transmitted infection worldwide (1), N. gonorrhoeae has developed resistance to every class of antibiotic used for treatment (2). In 2018, the World Health Organization (WHO) listed N. gonorrhoeae as a “priority pathogen” for which new treatments are urgently needed (3). The current treatment recommended by the WHO is ceftriaxone and azithromycin dual therapy, though several countries have transitioned to ceftriaxone monotherapy considering the increasing resistance to azithromycin (4–6). Ceftriaxone is the last available antibiotic for empirical treatment of N. gonorrhoeae. Several countries in Asia and Europe have reported >5% prevalence of strains with decreased susceptibility to ceftriaxone, the threshold used by the WHO to change the recommended therapy (7, 8).

Because the prevalence of antimicrobial resistance in N. gonorrhoeae continues to increase and our treatment options remain limited, it is critical to develop novel methods that detect ceftriaxone-resistant N. gonorrhoeae strains with the hope of slowing the spread of resistance. One promising method to accomplish this is through the development of molecular assays that predict antimicrobial resistance and can be used to guide treatment. For example, a molecular assay screening for the wild-type S91 amino acid in the *gyrA* gene of N. gonorrhoeae has been proven to screen effectively for ciprofloxacin susceptibility (9–11) and has allowed for resistance-guided therapy. In 2018, the UK national guidelines reinstated ciprofloxacin as the recommended treatment of N. gonorrhoeae when the susceptibility is known (12).

A molecular assay for ceftriaxone resistance is not yet available, largely due to the multiple mechanisms of resistance that N. gonorrhoeae employs, which primarily involve, but are not limited to, the four following genes: *penA*, *ponA*, *penB*, and *mtrR* (13–16). *penA* encodes the penicillin-binding protein (PBP) 2 for which mutations in at least six specific amino acid positions have been associated with decreased ceftriaxone susceptibility: A311V, A501V/P/T, N512Y, A516G, G542S, and G545S (described below) (2). *ponA* encodes the similar, yet less significant with respect to ceftriaxone resistance, PBP1, in which the L421P amino acid alteration is associated with increased MICs to β-lactam drugs (16). *penB* encodes the major outer membrane porin PorB, and amino acid alterations at G120 and A121 result in decreased permeability to antimicrobials (17). Lastly, *mtrR* encodes the transcriptional repressor of the MtrC-MtrD-MtrE efflux pump, and deletion of an adenine residue from the promoter region’s 13-bp inverted repeat results in increased flux of antimicrobials out of the cell, resulting in increased MICs (18).

One molecular assay in 2015 was developed to predict decreased susceptibility to ceftriaxone in Canadian strains, reporting sensitivities and specificities as high as 98.3% and 66.7%, respectively, by screening alleles in *penA*, *ponA*, *porB*, and *mtrR* (19). An updated multiplex molecular assay using the same four genes to predict intermediate-to-decreased susceptibility of Canadian strains to ceftriaxone achieved a sensitivity and specificity of 99.8% and 89.0%, respectively (20). While those results are promising, testing and validation were confined to a subset of Canadian N. gonorrhoeae strains, which might limit their generalizability (21). When applied to a global collection of genetic data, the combination of genetic markers decreased to a specificity of 67.3% (21). Assays based on diverse genomes from a global collection of N. gonorrhoeae isolates could enhance generalizability.

We previously designed four molecular algorithms for predicting decreased ceftriaxone susceptibility using at least one of *penA*, *ponA*, *porB*, and *mtrR* in a global collection of 3821 N. gonorrhoeae genomes, obtaining sensitivities and specificities up to 95% (with 62% specificity) and 72% (with 89% sensitivity), respectively (13). The algorithms differ in whether they utilized *penA* or non-*penA* genetic loci and whether mosaicism determination was performed. Those algorithms have similar sensitivity and specificity values to the assays developed by Peterson et al. (20) and may have wider applicability because they are based on genomic data from 23 countries. The algorithms, thus far, have proved successful in predicting decreased ceftriaxone susceptibility in novel resistant isolates (22) along with larger sets of isolates in specific countries (23). However, given the extensive genotypic variability of N. gonorrhoeae (24), further validation of these algorithms is needed within larger and more diverse sets of genomes, and additional work is required to develop global molecular assays with higher sensitivity and specificity combinations.

PathogenWatch (https://pathogen.watch/) is a public, online database to support genomic epidemiology and has both genomic data and metadata (including antimicrobial resistance [AMR] and MIC values) for over 12,000 N. gonorrhoeae strains. These sequences were quality-checked and assembled from available public archives (25). Therefore, the PathogenWatch database is one of the largest publicly available databases for N. gonorrhoeae genomic sequencing data. To further evaluate genetic markers, algorithms, and targets for molecular assays associated with decreased ceftriaxone susceptibility and resistance, the objective of this study was to use a computational approach to determine the performance and compare the results to prior estimates of each among the PathogenWatch database, the largest and most diverse public repository of N. gonorrhoeae whole-genome sequences and associated metadata to date (25).

## RESULTS

### Geographical distribution of N. gonorrhoeae in PathogenWatch database.

In total, there were 9,540 N. gonorrhoeae genomes obtained from the PathogenWatch database. The geographical distribution of those isolates is shown in Fig. S1 and Table S1. Overall, the N. gonorrhoeae isolates in the database were from 64 different countries with over 75% of the isolates originating from the following five countries in decreasing order: United States (*n* = 2,749; 28.8%), Australia (*n* = 2,220; 23.3%), United Kingdom (*n* = 1,394; 14.6%), Norway (*n* = 897; 9.4%), and New Zealand (*n* = 378; 4.0%).

Among the 9,540 N. gonorrhoeae isolates, 368 (4%) were categorized as having decreased susceptibility to ceftriaxone. The strains with decreased susceptibility were from 27 countries, as shown in Fig. S2 and Table S2. The most frequent isolates with decreased susceptibility were from the United States (*n* = 124; 33.7%), Canada (*n* = 75; 20.4%), Vietnam (*n* = 48; 13.0%), Japan (*n* = 36; 9.8%), and Norway (*n* = 16; 4.3%).

### Evaluating genetic mutations for predicting ceftriaxone resistance.

**FIG 1:**
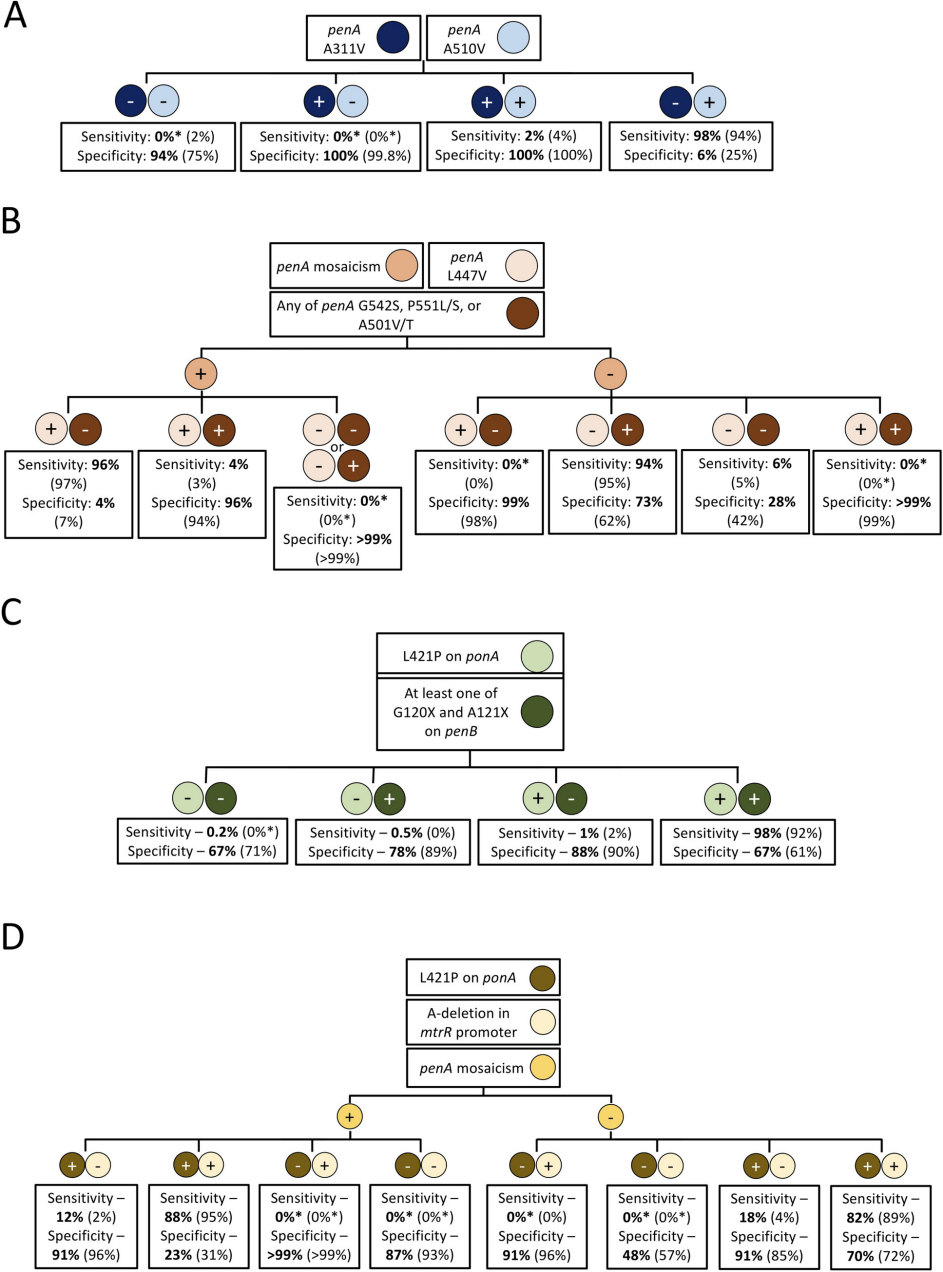
Four molecular algorithms with sensitivity and specificity values using published genetic data on (i) PathogenWatch (bolded numbers, this work) and (ii) previously reported in a global collection of genetic data (numbers in parentheses, previous work) (13). (+) indicates the presence of the genetic alteration, while (−) indicates the absence thereof. Sensitivity and specificity values are for ceftriaxone's decreased susceptibility. All genetic loci of each algorithm are to be tested simultaneously for, and not stepwise. Asterisks denote that no strains with decreased susceptibility were reported for that specific combination of genetic alterations.

Sensitivity and specificity values for molecular markers implicated in ceftriaxone resistance in N. gonorrhoeae are listed in Table 1 alongside comparisons to prior reports that analyzed these loci in predicting decreased ceftriaxone susceptibility (13, 19). The four molecular algorithms we previously designed are shown in Fig. 1 and Table S3 with new sensitivity and specificity values when applied to the PathogenWatch database in bold (13). The analysis using the PathogenWatch database resulted in similar sensitivity and specificity values as the prior molecular markers, assays, and algorithms of interest. For example, having at least 1 alteration in either G120 or A121 of *porB* resulted in a 98.4% sensitivity and 44.6% specificity, which was in comparison to 96.6% and 42.1% sensitivity and specificity, respectively, found in our prior work (13) and 94.8% and 50.8% sensitivity and specificity, respectively, by Peterson et al. (19). Having no *penA* mosaicism, adenine deletion in the promoter region of *mtrR*, and L421P in *ponA* as shown in the Fig. 1D algorithm resulted in an 82% sensitivity and 70% specificity, in comparison to the 89% sensitivity and 72% specificity found previously (13).

**TABLE 1:** Sensitivity and specificity values of applying various genetic alterations contributing to ceftriaxone resistance in N. gonorrhoeae to the PathogenWatch database and comparison with previous studies

Genetic mutations	Parameter	PathogenWatch	Prior studies
MtrR promoter del	Sensitivity	88.9%	95.3%^*a*^, 98.3%^*b*^
	Specificity	62.9%	52.8%^*a*^, 65.2%^*b*^
*porB* at least 1 of G120X, A121X	Sensitivity	98.4%	96.6%^*a*^, 94.8%^*b*^
	Specificity	44.6%	42.1%^*a*^, 50.8%^*b*^
*porB* both G120X, A121X	Sensitivity	96.2%	94.3%^*a*^
	Specificity	61.2%	52.3%^*a*^
*ponA* L421P	Sensitivity	99.2%	99.6%^*a*^, 100%^*b*^
	Specificity	55.7%	45.4%^*a*^, 57.4%^*b*^
*penA* A311V	Sensitivity	2.4%	3.9%^*a*^
	Specificity	100.0%	99.9%^*a*^
*penA* A501P/V/T	Sensitivity	27.4%	36.9%^*a*^
	Specificity	93.0%	93.6%^*a*^
*penA* N512Y	Sensitivity	61.4%	42.0%^*a*^
	Specificity	89.1%	75.8%^*a*^
*penA* A516G	Sensitivity	38.6%	55.0%^*a*^
	Specificity	17.3%	30.5%^*a*^
*penA* G542S	Sensitivity	12.8%	19.3%^*a*^
	Specificity	93.2%	86.3%^*a*^
*penA* G545S	Sensitivity	61.4%	41.8%^*a*^
	Specificity	90.1%	85.9%^*a*^
Peterson et al. (20) assay. Any three of: *ponA* L421P, at least 1 of *porB* G120X and A121X, *mtrR* −35delA, *penA* A311V, *penA* A501, *penA* N512Y, *penA* G545S	Sensitivity	96.5%	99.8%
	Specificity	70.3%	89.0%

a Lin et al. (13).

b Peterson et al. (19) with the definition of decreased ceftriaxone susceptibility as ≥0.06 mg/liter as opposed to >0.064 mg/liter in our original work (13) and here.

### Modeling predictive values with respect to resistance prevalence.

**FIG 2:**
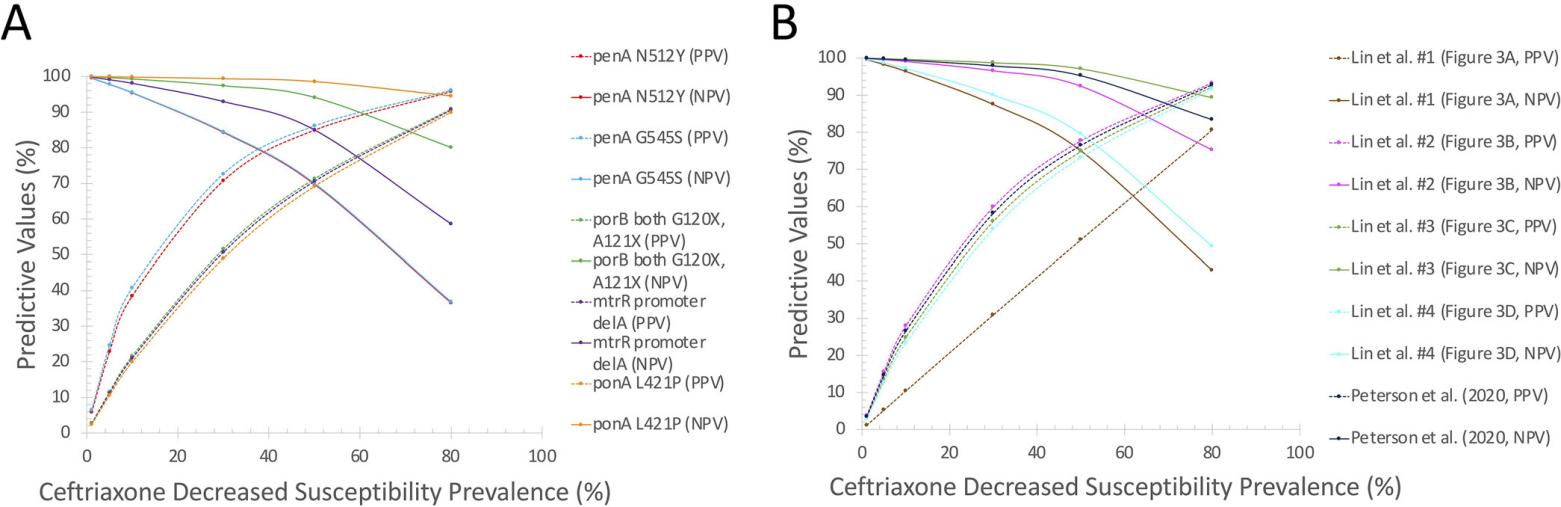
Graphs of the positive and negative predictive values (PPV and NPV, respectively) for prediction of ceftriaxone decreased susceptibility by differing prevalence of decreased ceftriaxone susceptibility. (A) The five molecular markers with the highest sensitivity and specificity combinations for decreased ceftriaxone susceptibility. (B) The five algorithms or assays with the highest sensitivity and specificity combinations for decreased ceftriaxone susceptibility.

To gauge the potential clinical utility of the molecular assays, algorithms, and markers within the PathogenWatch database, the positive and negative predictive values of the molecular markers, algorithms, and assays with the highest sensitivity and specificity values were estimated using the different prevalence of decreased ceftriaxone susceptibility as shown in Fig. 2 and Table S4 and S5. For example, with a prevalence of decreased ceftriaxone susceptibility of 1.1%, the positive and negative predictive values of L421P in *ponA* and at least one alteration in G120, A121 in *porB* (Fig. 1C algorithm), were 99.9% and 3.2%, respectively. At a prevalence of 10%, the positive predictive value decreased to 99.7%, and the negative predictive value increased to 24.8%.

Single amino acid alterations of one gene often exhibited high positive or negative predictive values with low complementary negative or positive predictive values, respectively. For example, *ponA* L421P has the highest negative predictive value at all prevalence values (e.g., 98.6% at 50% prevalence) but has the second-lowest positive predictive values (e.g., 69.1% at 50% prevalence). In contrast, the targets shown in Fig. 1B resulted in the second-highest positive predictive values (77.7% at 50% prevalence) and 5th highest negative predictive values (92.4% at 50% prevalence), whereas the targets reported by Peterson et al. (19) were associated with the 3rd highest positive and negative predictive values (76.5% and 95%, respectively, at 50% prevalence).

## DISCUSSION

Using the PathogenWatch platform, we used a computational approach to evaluate several genetic mutations from previously reported algorithms and assays to predict decreased susceptibility to ceftriaxone within a global collection of 9,540 N. gonorrhoeae genomes. Our study found an overall consistency in the sensitivity and specificity values for several genetic markers, algorithms, and assays used to predict decreased ceftriaxone susceptibility in this diverse collection of N. gonorrhoeae genomes. The sensitivity and specificity values were used to model the positive and negative predictive values according to the different prevalence of decreased susceptibility to ceftriaxone, which can be used in different testing scenarios. Our findings support the potential viability of molecular assays to predict globally decreased susceptibility to ceftriaxone. Those assays will be of greater utility in settings with a high prevalence of decreased susceptibility to ceftriaxone.

Overall, all genetic loci were found to have a general concordance in sensitivity and specificity values with slightly higher variance in specificity values. For example, the presence of either G120X and A121X in *porB* within the PathogenWatch data was within 3% in both sensitivity and specificity. Some mutations, like A501P/V/T in *PenA*, were associated with high concordance in specificity (93.0% versus 93.6%) but differed in sensitivity (27.4% versus 36.9%), while other mutations, such as having alterations in both G120 and A121 of *porB*, were associated with high concordance in sensitivity (96.2% versus 94.3%) but differed to a greater extent in specificity (61.2% versus 52.3%). With respect to the molecular algorithms and assays, the presence of L421P in *ponA* and at least one of G120X and A121X in *porB* resulted in a 98% and 67% sensitivity and specificity here, which was similar to the 92% and 61% sensitivity and specificity previously obtained (13). Applying the molecular targets reported by Peterson et al. (20) to the PathogenWatch database resulted in a slight decrease in sensitivity (96.5% versus 99.8%), but a larger decrease in specificity (70.3% versus 89.0%).

Slight variations in the sensitivity and specificity values were expected due to the increased diversity of genomes included in the PathogenWatch database. Based on the global collection of isolates compiled from published articles detailed in our previous work (13), the United States similarly comprised most isolates (30%), but the following countries had the next highest quantities of isolates in that report: Russia (14%), Canada (12%), New Zealand (11%), and China (8%) (13). Moreover, the PathogenWatch database contains genomic data from 64 countries in contrast to the 23 countries included in our previous report (13). The similarities in sensitivity and specificity values despite the expanded geographical diversity highlight the potential utility of designing a molecular assay to predict ceftriaxone resistance globally. Moreover, the geographic variation in genetic markers of resistance is expected given the heterogeneity in mechanisms contributing to ceftriaxone resistance in N. gonorrhoeae. While many of these alterations are important underlying mechanisms of resistance as indicated by their high sensitivity or specificity values, the combination of these alterations possessed by resistant strains may vary from region to region, leading to variation in sensitivity and specificity values and making the development of a global molecular assay difficult. However, given the rising spread of ceftriaxone-resistant strains internationally, a molecular assay that can be used globally is necessary.

Despite the genetic variation, the preliminary results from this work still demonstrated promise for the identification of genetic markers to predict ceftriaxone resistance. Comparing our sensitivity and specificity values to the assay used by Peterson et al. (99.8%, 89.0%, respectively) (20), we obtained a similarly high sensitivity with a lower specificity of 96.5% and 70.3%, respectively. Moreover, while several of these markers may have been validated in some of our previous works (13, 22, 23), it is critical to validate their robustness in several different populations due to the wide genotypic variability of N. gonorrhoeae that arises from its extraordinary ability for genetic recombination and acquisition of mutations (26). We previously reported how the Peterson et al. (21) assay was associated with a greater than 20% decrease in specificity when applied to another global set of isolates. Validating the genetic markers against 9,540 isolates from 64 countries is highly promising because this is the largest set of isolates that have been used to validate the above genetic markers and algorithms. With further refinement and work toward pursuing a global assay, it is foreseeable to achieve the optimal combination of genetic alterations with higher sensitivity and specificity values. As global surveillance of N. gonorrhoeae and antimicrobial resistance expands alongside whole-genome sequencing of all isolates, molecular prediction assays can continue to be refined based on genomic epidemiology (27).

Importantly, this is the first work in which we have modeled the positive and negative predictive values with respect to the prevalence of decreased ceftriaxone susceptibility prevalence in different settings. From our analysis, we outlined different combinations of genetic mutations and different scenarios needed to obtain high positive and negative predictive values in predicting ceftriaxone's decreased susceptibility. At a 1.1% prevalence as reported by an sexually transmitted infection (STI) clinic in Amsterdam (28), all the molecular markers resulted in extremely high negative predictive values >99% with positive predictive values <10%. In populations like those, molecular assays might not be useful given the high proportion of false-positive to true positive tests. However, in areas of China where estimates of the prevalence of decreased ceftriaxone susceptibility approaches 30% (29), the positive predictive values of those assays are nearly 60% while maintaining high negative predictive values. For example, one of the algorithms in our prior work using *penA* mosaicism, *PenA* L447V, and any of G542S, P551L/S, or A501V/T corresponded with 59.9% and 96.6% positive and negative predictive values, respectively, while using any three of the seven genetic targets in the Peterson et al. (20) assay corresponded with a 58.2% positive predictive value and 97.9% negative predictive value (13, 20). In contrast, single genetic mutations, such as *PenA* N512Y and G545S, are also associated with an increase in positive predictive values but a decrease in negative predictive values <90%. Moreover, while the positive predictive values of the molecular algorithms leave room for a significant portion of false-positives, the utility of these tests will continue to grow as the prevalence further increases.

One of the limitations of our findings is the relatively low prevalence (4%) of strains with decreased susceptibility to ceftriaxone publicly available in the PathogenWatch database. The low prevalence might cause less precise sensitivities or may lead to decreased global generalizability. Still, the sensitivity values reported here were consistent with those of prior reports, and we expect the estimates to improve as more decreased susceptible strains undergo whole-genome sequencing. Another limitation is the inherent lack of standardization of MIC data. While many sequences that include MIC data are from prior peer-reviewed data or from government public health laboratories that have described their antimicrobial susceptibility testing methods, there is currently no method to prove the quality of MIC data used in the database. A third limitation is that the global distribution of N. gonorrhoeae isolates currently present in PathogenWatch were not reflective of the true global distribution of isolates. Instead, they are mostly from high-resourced countries, reflecting the distribution of resources that enable extensive testing, sequencing, and subsequent publishing of N. gonorrhoeae genomic data. Therefore, while this work provides further support on the viability of global assay in predicting decreased ceftriaxone susceptibility, further evaluation on a more representative global set of isolates is needed. This can be achieved with an extensive expansion of public health and genomic surveillance measures of AMR in N. gonorrhoeae in lower-resourced settings where resistance might be higher (30).

### Conclusion.

Assays to detect resistance to ceftriaxone in N. gonorrhoeae on a global scale are critically needed. Our study found consistent sensitivity and specificity values when applying different genetic alterations and previously reported molecular algorithms and assays within a global collection of N. gonorrhoeae genomes obtained from PathogenWatch, providing plausibility and support for the development of globally precise molecular assays that can be used to predict decreased susceptibility to ceftriaxone. As the prevalence of resistance to ceftriaxone in N. gonorrhoeae increases, so too will the potential utility of current and future molecular assays.

## MATERIALS AND METHODS

### Whole-genome sequence data.

All 12,943 publicly available N. gonorrhoeae whole-genome sequences and associated metadata (MIC values, antibiotic susceptibility classification, country of origin) were extracted from the PathogenWatch database on November 17, 2020 and used in the analysis (31).

### Mutation analysis.

Four genes (*penA*, *ponA*, *porB*, and *mtrR*) were downloaded from the EzBioCloud database using the N. gonorrhoeae FA 1090 strain as a reference (32). Using 60% identity/length as a threshold, a Basic Local Alignment Search Tool (BLAST v2.2.26+) search was performed to query each of the four genes against the N. gonorrhoeae genomes downloaded from PathogenWatch (33). The results were parsed using the Biopython BLAST IO package, followed by the translation of the DNA to protein (34). An in-house python code that counts the frequency of different amino acids within a given position value was used to identify all mutations of interest (available at https://github.com/smha118/mutation_detecter).

The promoter region for *mtrR* was obtained by extracting upper 60 bp nucleotides (nt) from the start codon position in the BLAST result file. Because our target mutation for the promoter region is deletion, the length of promoter sequences was varied. Thus, to check deletion with respect to the reference, multiple sequence alignment was conducted. Promoter regions were clustered and categorized based on exact match identity with other promoter sequences. Only one representative sequence was collected from each cluster. This was done to bring down the number of sequences to be aligned as the alignment requires high computing memories. Multiple sequence alignment was performed with MUSCLE v3.8.31 (35).

From the PathogenWatch database, 12,943 N. gonorrhoeae strains were initially identified. Strains without reported MIC and susceptibility data were excluded from the study (*n* = 2,995), leaving 9,948 N. gonorrhoeae isolates. Due to the ambiguity of decreased ceftriaxone susceptibility status, which we defined as >0.064 mg/liter (*n* = 46) as commonly classified by other investigators (36–39), isolates were excluded if they (i) were categorized without exact MIC values according to a MIC cutoff of 0.125 mg/liter (*n* = 362) or (ii) had MIC value ranges that overlapped with the decreased susceptibility cutoff. For example, isolates ERR3623188 and ERR3623235 used a MIC cutoff of ≥0.125 mg/liter, and isolates SRR8566948 and SRR8566968 had a MIC range of 0.06 to 0.125 mg/liter. In total, there were 9540 isolates with metadata, MIC or susceptibility data, and genetic data available for analysis.

### Genetic alterations important for ceftriaxone resistance.

A multivariate regression model to predict the MIC values of ceftriaxone in N. gonorrhoeae was previously designed based on the genetic alterations that influence ceftriaxone MIC: (i) *penA*: A311V, A501P/T/V, N512Y, A516G, and G542S; (ii) *porB*: any change at G120; (iii) *ponA*: L421P; and (iv) *mtrR*: promoter disruption (e.g., −35A deletion). The alterations with the highest impact on ceftriaxone MICs were A501P/T/V, A311V, N512Y in *penA*, and G120 alteration in *porB* (R^2^ = 0.721) (40). Those mutations were used in our analysis. While A121 alteration in *porB* was not included in the regression model, we included it because it is associated with increased ceftriaxone resistance in N. gonorrhoeae (41).

### Analysis.

The whole-genome sequences from PathogenWatch were first analyzed for the presence or absence of the nine specific genetic alterations mentioned above. The N. gonorrhoeae genomes within the PathogenWatch data set were then evaluated against previous molecular algorithms and assays predicting decreased ceftriaxone susceptibility, specifically the molecular assay proposed by Peterson et al. (20) and the four algorithms designed in our previous work (13). For each genetic marker, algorithm, and assay of interest, the sensitivities, specificities, positive predictive values, and negative predictive values were calculated (42, 43). Sensitivity was defined as the number of isolates with the genotype of interest and decreased ceftriaxone susceptibility divided by the total number of isolates with decreased ceftriaxone susceptibility. Specificity was defined as the number of isolates without the genotype of interest and decreased ceftriaxone susceptibility divided by the total number of isolates without decreased ceftriaxone susceptibility. The positive predictive value was the number of isolates with the genotype and decreased ceftriaxone susceptibility divided by the total number of isolates with the genotype. The negative predictive value was the number of isolates without the genotype and decreased ceftriaxone susceptibility divided by the total number of isolates without the genotype. While sensitivity and specificity values in this work were useful as general indicators of the accuracy of the genetic markers in predicting decreased ceftriaxone susceptibility, positive and negative predictive values were more useful in indicating the utility of those genetic markers by considering the prevalence of decreased ceftriaxone susceptibility within a population of interest.

### Data availability.

All N. gonorrhoeae genomes used in this study are at the PathogenWatch database (https://pathogen.watch/genomes/all?genusId=482&speciesId=485). The code used to detect all genetic mutations is available at https://github.com/smha118/mutation_detecter.
