# Supplementary material for: Reliability of Genetic Alterations in Predicting Ceftriaxone Resistance in Neisseria gonorrhoeae Globally
Source: Microbiol Spectr. 2022 Mar 29;10(2):e02065-21. doi: 10.1128/spectrum.02065-21 (PMC9045316; doi:10.1128/spectrum.02065-21)
Supplement: SUPPLEMENTAL FILE 1 — Supplemental material. Download SPECTRUM02065-21_Supp_1_seq7.pdf, PDF file, 1.6 MB [file spectrum02065-21_supp_1_seq7.pdf]

Supplementary Information

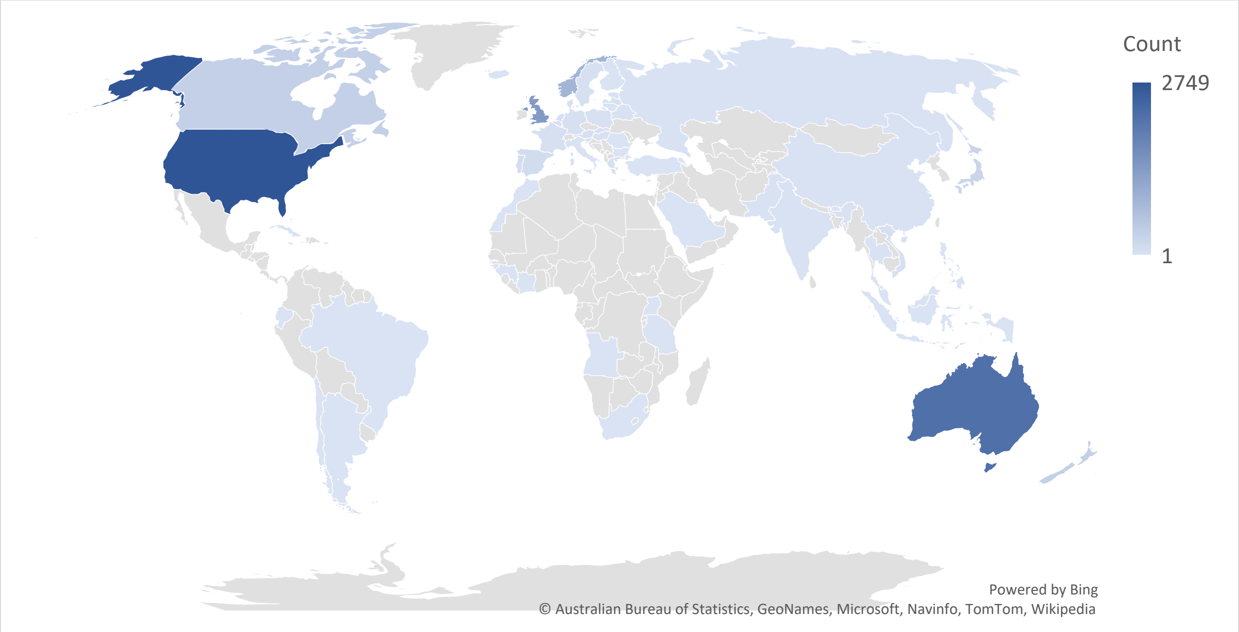

Figure S1. Heat Map of World Distribution of *N. gonorrhoeae* strains with ceftriaxone MIC and susceptibility data in the PathogenWatch database

Table S1. World Distribution of *N. gonorrhoeae* strains with ceftriaxone MIC and susceptibility data in the PathogenWatch database

Country	# of strains	% of total
United States	2749	28.8
Australia	2220	23.3
United Kingdom	1394	14.6
Norway	897	9.4
New Zealand	378	4.0
Canada	365	3.8

Japan	264	2.8
Spain	129	1.4
Portugal	108	1.1
Slovenia	77	0.8
Netherlands	71	0.7
Denmark	63	0.7
France	62	0.6
Belgium	55	0.6
Austria	54	0.6
Greece	54	0.6
Germany	53	0.6
Vietnam	52	0.5
Sweden	51	0.5
Hungary	48	0.5
Slovakia	39	0.4
Latvia	38	0.4
Poland	34	0.4
Italy	28	0.3
India	24	0.3
Belarus	23	0.2
Guinea-Bissau	22	0.2
Thailand	22	0.2

Malta	20	0.2
China	18	0.2
Estonia	17	0.2
Pakistan	14	0.1
Philippines	14	0.1
Russia	13	0.1
Cyprus	8	0.1
Bhutan	7	0.1
Gambia	5	0.1
Iceland	5	0.1
Indonesia	5	0.1
Cuba	4	<0.1
Turkey	4	<0.1
Morocco	3	<0.1
South Africa	3	<0.1
Brasil	2	<0.1
Cabo Verde	2	<0.1
Ivory Coast	2	<0.1
Scotland	2	<0.1
Tanzania	2	<0.1
Angola	1	<0.1
Argentina	1	<0.1

Armenia	1	<0.1
Bulgaria	1	<0.1
Caribbean	1	<0.1
Chile	1	<0.1
Ecuador	1	<0.1
Finland	1	<0.1
Guinea	1	<0.1
Hong Kong	1	<0.1
Jamaica	1	<0.1
Lithuania	1	<0.1
Malaysia	1	<0.1
Romania	1	<0.1
Saudi Arabia	1	<0.1
Uganda	1	<0.1

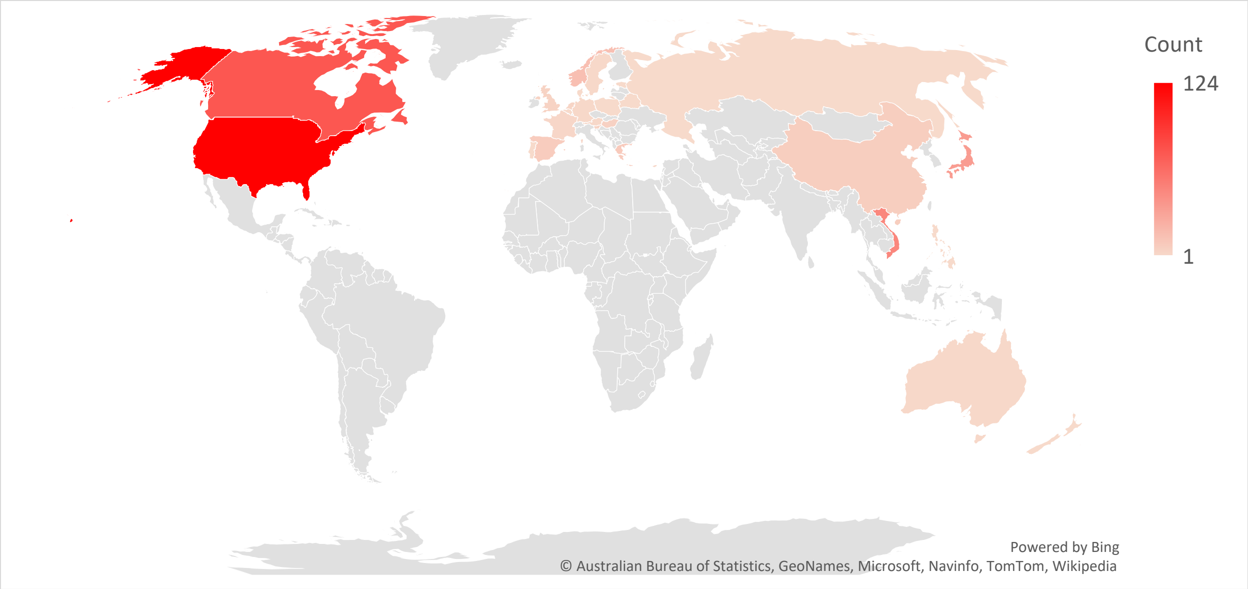

Figure S2. Heat Map of World distribution of decreased susceptible strains of *N. gonorrhoeae* in the PathogenWatch database.

Table S2. World distribution of decreased susceptible strains of *N. gonorrhoeae* in the PathogenWatch database.

Country	# of strains	% of total
United States	124	33.7
Canada	75	20.4
Vietnam	48	13.0
Japan	36	9.8
Norway	16	4.3
Greece	10	2.7
Spain	9	2.4
China	8	2.2

Hungary	6	1.6
Slovenia	5	1.4
United Kingdom	5	1.4
Netherlands	4	1.1
France	3	0.8
Australia	2	0.5
Austria	2	0.5
Cyprus	2	0.5
Germany	2	0.5
Sweden	2	0.5
Belarus	1	0.3
Belgium	1	0.3
Denmark	1	0.3
Estonia	1	0.3
New Zealand	1	0.3
Philippines	1	0.3
Poland	1	0.3
Portugal	1	0.3
Russia	1	0.3

Table S3. Comparison of sensitivity and specificity values of four molecular algorithms detailed in previous study when using published genetic data on 1) PathogenWatch (bolded

numbers, this work) and 2) the original global collection of genetic data used to formulate the algorithms (non-bolded numbers, previous work).(13) A1 denotes algorithm #1, A2 indicates algorithm #2, and so on. (+) indicates presence of the genetic alteration, while (-) indicates absence thereof. Sensitivity and specificity values are for ceftriaxone decreased susceptibility. All genetic loci of each algorithm are to be tested simultaneously for, and not step-wise. Asterisks denote that no strains with decreased susceptibility were reported for that specific combination of genetic alterations.

**Genetic Mutations**	**Current study (n=9540)**		Previous study (n=3821)	
	**Sensitivity**	**Specificity**	Sensitivity	Specificity
**A1**: (-) *penA* A311V, (-) *penA* A510V	**0%***	**4%**	2%	75%
**A1**: (+) *penA* A311V, (-) *penA* A510V	**0%***	**100%**	0%*	99.8%
**A1**: (+) *penA* A311V, (+) *penA* A510V	**2%**	**100%**	4%	100%
**A1**: (-) *penA* A311V, (+) *penA* A510V	**98%**	**6%**	94%	25%
**A2**: (+) *penA* mosaicism, (+) *penA* L447V, (-) Any of *penA* G542S, P551L/S, or A501V/T	**96%**	**4%**	97%	7%
**A2**: (+) *penA* mosaicism, (+) *penA* L447V, (+) Any of *penA* G542S, P551L/S, or A501V/T	**4%**	**96%**	3%	94%
**A2**: (+) *penA* mosaicism, (-) *penA* L447V, (+/-) Any of *penA* G542S, P551L/S, or A501V/T	**0%***	**>99%**	0%*	>99%

**A2**: (-) *penA* mosaicism, (+) *penA* L447V, (-) Any of *penA* G542S, P551L/S, or A501V/T	**0%***	**99%**	0%	98%
**A2**: (-) *penA* mosaicism, (-) *penA* L447V, (+) Any of *penA* G542S, P551L/S, or A501V/T	**94%**	**73%**	95%	62%
**A2**: (-) *penA* mosaicism, (-) *penA* L447V, (-) Any of *penA* G542S, P551L/S, or A501V/T	**6%**	**28%**	5%	42%
**A2**: (-) *penA* mosaicism, (+) *penA* L447V, (+) Any of *penA* G542S, P551L/S, or A501V/T	**0%***	**>99%**	0%*	99%
**A3**: (-) *ponA* L421P, (-) At least one of *penB* G120X and A121X	**0.2%**	**67%**	0%*	71%
**A3**: (-) *ponA* L421P, (+) At least one of *penB* G120X and A121X	**0.5%**	**78%**	0%	89%
**A3**: (-) *ponA* L421P, (-) At least one of *penB* G120X and A121X	**1%**	**88%**	2%	90%
**A3**: (-) *ponA* L421P, (-) At least one of *penB* G120X and A121X	**98%**	**67%**	92%	61%
**A4**: (+) *penA* mosaicism, (+) *ponA* L421P, (-) *mtrR* promoter A-deletion	**12%**	**91%**	2%	96%

**A4**: (+) *penA* mosaicism, (+) *ponA* L421P, (+) *mtrR* promoter A-deletion	**88%**	**23%**	95%	31%
**A4**: (+) *penA* mosaicism, (-) *ponA* L421P, (+) *mtrR* promoter A-deletion	**0%***	**>99%**	0%*	>99%
**A4**: (+) *penA* mosaicism, (-) *ponA* L421P, (-) *mtrR* promoter A-deletion	**0%***	**87%**	0%*	93%
**A4**: (-) *penA* mosaicism, (-) *ponA* L421P, (+) *mtrR* promoter A-deletion	**0%***	**91%**	0%	96%
**A4**: (-) *penA* mosaicism, (-) *ponA* L421P, (-) *mtrR* promoter A-deletion	**0%***	**48%**	0%*	57%
**A4**: (-) *penA* mosaicism, (+) *ponA* L421P, (-) *mtrR* promoter A-deletion	**18%**	**91%**	4%	85%
**A4**: (-) *penA* mosaicism, (+) *ponA* L421P, (+) *mtrR* promoter A-deletion	**82%**	**70%**	89%	72%

Table S4. Positive predictive values (%) of molecular markers, algorithms, and assays with the highest sensitivity and specificity combinations with respect to prevalence of ceftriaxone decreased susceptibility.

**Prevalence (%)**	**Lin et al. #1 (Figure 3A)**	**Lin et al. #2 (Figure 3B)**	**Lin et al. #3 (Figure 3C)**	**Lin et al. #4 (Figure 3D)**	**Peterson et al. (2020)**	***ponA* L421P**	***penA* N512Y**	***penA* G545S**	***porB* both G120 and A121**	***mtrR* promoter A-deletion**

**1.1**	1.1	3.7	3.2	3.0	3.5	2.4	5.9	6.5	2.7	2.6
**5**	5.2	15.5	13.5	12.6	14.6	10.5	22.9	24.6	11.5	11.2
**10**	10.4	27.9	24.8	23.3	26.5	19.9	38.5	40.8	21.6	21.0
**30**	30.9	59.9	56	54.0	58.2	49.0	70.7	72.7	51.5	50.7
**50**	51.0	77.7	74.8	73.2	76.5	69.1	84.9	86.1	71.3	70.6
**80**	80.7	93.3	92.2	91.6	92.9	90.0	95.8	96.1	90.8	90.6

Table S5. Negative predictive values (%) of molecular markers, algorithms, and assays with the highest sensitivity and specificity combinations with respect to prevalence of ceftriaxone decreased susceptibility.

**Prevalence (%)**	**Lin et al. #1 (Figure 3A)**	**Lin et al. #2 (Figure 3B)**	**Lin et al. #3 (Figure 3C)**	**Lin et al. #4 (Figure 3D)**	**Peterson et al. (2020)**	***ponA* L421P**	***penA* N512Y**	***penA* G545S**	***porB* both G120 and A121**	***mtrR* promoter A-deletion**
**1.1**	99.6	99.9	>99.9	99.7	99.9	>99.9	99.5	99.5	99.9	99.8
**5**	98.3	99.6	99.8	98.7	99.7	99.9	97.8	97.8	99.7	99.1
**10**	96.4	99.1	99.7	97.2	99.5	99.8	95.4	95.5	99.3	98.1
**30**	87.5	96.6	98.7	90.1	97.9	99.4	84.3	84.5	97.4	93.0
**50**	75	92.4	97.1	79.6	95.3	98.6	69.8	70.0	94.2	85.0
**80**	42.9	75.3	89.3	49.3	83.4	94.6	36.6	36.9	80.1	58.6
